# Supplementary material for: The interactome of intact mitochondria by cross-linking mass spectrometry provides evidence for coexisting respiratory supercomplexes
Source: Mol Cell Proteomics. 2017 Dec 8;17(2):216–32. doi: 10.1074/mcp.RA117.000470 (PMC5795388; doi:10.1074/mcp.RA117.000470)
Supplement: Supplemental Data [file supp_RA117.000470_133922_0_supp_23978_fzffwf.zip › spectra_annotation/mito_spectra_annotation/240-2-3-1-4-1.pdf]

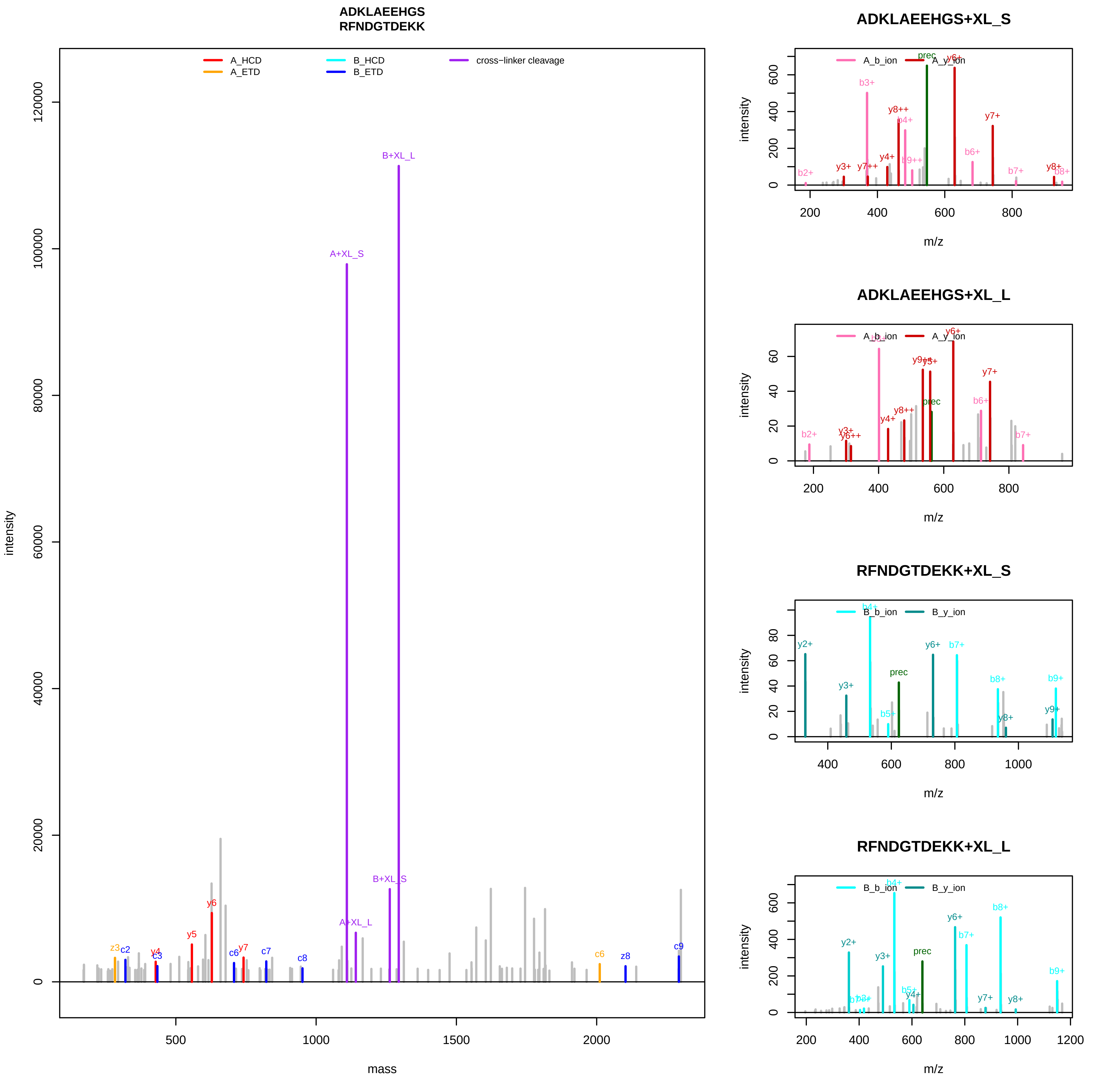
ADKLAEEHGS
RFNDGTDEKK
A_HCD
A_ETD
B_HCD
B_ETD
cross−linker cleavage
intensity
mass
0
20000
40000
60000
80000
100000
120000
500
1000
1500
2000
B+XL_L
A+XL_S
B+XL_S
A+XL_L
z3
c2
y4
c3
y5
y6
c6
y7
c7
c8
c6
z8
c9
ADKLAEEHGS+XL_S
A_b_ion
A_y_ion
prec
b2+
y3+
y7++
b3+
y4+
y8++
b4+
b9++
y6+
b6+
y7+
b7+
y8+
b8+
m/z
ADKLAEEHGS+XL_L
y3+
y6++
y9++
y5+
y8++
RFNDGTDEKK+XL_S
B_b_ion
B_y_ion
y2+
b4+
b5+
y6+
b7+
b8+
y8+
b9+
y9+
RFNDGTDEKK+XL_L
y4+
y7+
b9+
